# Evaluation of a microscale quantitative suspension test to determine the bactericidal and yeasticidal activity of glutaral – one step to improve sustainability in disinfectant testing

**DOI:** 10.3205/dgkh000458

**Published:** 2024-01-30

**Authors:** Jürgen Gebel, Marvin Rausch, Katja Bienentreu, Felix Droop, Maren Eggers, Lea Gebel, Stefanie Gemein, Britt Hornei, Carola Ilschner, Anja Jacobshagen, Günter Kampf, Cihan Papan, Kira Roesch, Luisa Schmitz, Miranda Suchomel, Lutz Vossebein, Nico T. Mutters, Martin Exner

**Affiliations:** 1 Institute for Hygiene and Public Health, University Clinics Bonn, Bonn, Germany; 2 VAH – Association for Applied Hygiene c/o Institute for Hygiene and Public Health, Bonn, Germany; 3 Laboratory Prof. Gisela Enders MVZ GbR, Stuttgart, Germany; 4 Referenzinstitut für Bioanalytik, Bonn, Germany; 5 Evangelisches Krankenhaus Oberhausen, Zentralbereich Krankenhaushygiene, Institut für Laboratoriumsmedizin und Klinische Mikrobiologie, Oberhausen; 6 Federal Institute of Drugs and Medical Devices (BfArM) – Medical Devices Division, Bonn, Germany; 7 University Medicine Greifswald, Greifswald, Germany; 8 Institute for Hygiene and Applied Immunology, Medical University Vienna, Medical-technical Hygiene, Vienna, Austria; 9 Hochschule Niederrhein – Fachbereich Textil- und Bekleidungstechnik, Mönchengladbach, Germany

**Keywords:** suspension test, bactericidal activity, yeasticidal activity, micromethod, microscale suspension test, sustainability, glutaral

## Abstract

**Aims:**

To evaluate a newly developed microscale quantitative suspension test compared to the existing standard suspension test using determination of the bactericidal and yeasticidal activity of glutaral as one step to improve the sustainability of disinfectant testing.

**Methods:**

The testing principles of the quantitative suspension test according to VAH method 9 (comparable to EN 13727) was used as a standard suspension test using 8.0 mL product test solution, 1.0 mL organic load and 1.0 mL test suspension. In addition, a micro-scale suspension test was performed in 96-well plates with 160 µL product test solution, 20 µL organic load and 20 µL test suspension. *S. aureus* ATCC 6538, *P. aeruginosa* ATCC 15442 and *C. albicans* ATCC 10231 were test organisms. Glutaral was tested at concentrations of 0.05%, 0.1%, 0.2% and 0.3% with exposure times of 1, 5 and 15 min. Polysorbate 80 (30 g/L), lecithin (9 g/L), L-histidine (1 g/L) and glycine (10 g/L) were used as validated neutralizers. After serial dilution of the disinfectant-neutralizer-mixture, plates were incubated for 48 h at 36°C (bacteria) or 72 hours at 30°C (*C. albicans*) and colony forming units (cfu) counted. The lg reduction was calculated as the difference between the results of the water control and the disinfectant at the end of the exposure time. All experiments were done in triplicate under clean conditions. Means of lg reduction were compared with the unpaired t-test, p<0.05 was considered to be significant.

**Results:**

Sufficient bactericidal activity according the VAH test requirements of at least 5 lg was found with both methods in 16 data sets of 24 data sets in total, and insufficient bactericidal activity of less than 5 lg was found with both methods in 7 data sets. In one data set, the mean lg reduction was above 5 lg with the microscale method and <5 lg with the VAH method, with no significant difference between the data sets (p=0.3096; 0.2% glutaral, 1 min, *P. aeruginosa*). A sufficient yeasticidal activity of at least 4 lg was found with both methods in one data set, an insufficient yeasticidal activity of less than 4 lg was found with both methods in 8 data sets. With one exception, no significant differences were detected between the two methods below the efficacy threshold.

**Conclusions:**

The microscale quantitative suspension test proved to provide results similar to those of VAH method 9 when the bactericidal and yeasticidal activity of glutaralwas evaluated, with 32 out of 33 evaluations yielding consistent results in terms of efficacy. Its suitability should be confirmed with additional bacterial species, additional biocidal active substances and in other laboratories.

## Zusammenfassung

**Zielsetzung:**

Evaluierung eines neu entwickelten Mikro-Suspensionstests im Vergleich zur bisherigen Standardmethode am Beispiel der Bestimmung der bakteriziden und levuroziden Wirkung von Glutaral als ein Schritt auf dem Weg zu mehr Nachhaltigkeit in der Desinfektionsmittel-Testung.

**Methode:**

Die VAH-Methode 9 wurde als Standard-Suspensionstest mit 8,0 mL Produkttestlösung, 1,0 mL organischer Belastung und 1,0 mL Testsuspension verwendet. Darüber hinaus wurde ein Mikroskala-Suspensionstest (Mikromethode) in 96-Well-Platten mit 160 µL Produkttestlösung, 20 µL organischer Belastung und 20 µL Testsuspension durchgeführt. Als Testorganismen dienten *S. aureus* ATCC 6538, *P. aeruginosa* ATCC 15442 und *C. albicans* ATCC 10231. Glutaral wurde in Konzentrationen von 0,05%, 0,1%, 0,2% und 0,3% mit Expositionszeiten von 1, 5 und 15 min getestet. Polysorbat 80 (30 g/L), Lecithin (9 g/L), L-Histidin (1 g/L) und Glycin (10 g/L) wurden als validierte Neutralisationssubstanzen verwendet. Nach serieller Verdünnung des Desinfektionsmittel-Neutralisator-Gemischs wurden die Platten 48 h bei 36°C (Bakterien) bzw. 72 h bei 30°C (*C. albicans*) bebrütet und die Kolonie bildenden Einheiten (KbE) gezählt. Die lg-Reduktion wurde als Differenz zwischen den Ergebnissen der Wasserkontrolle und des Desinfektionsmittels am Ende der Expositionszeit berechnet. Alle Experimente wurden in dreifacher Ausführung bei geringer Belastung durchgeführt. Die Mittelwerte der lg-Reduktion wurden mit dem ungepaarten t-Test verglichen, wobei ein p-Wert <0,05 als signifikant angesehen wurde.

**Ergebnisse:**

Eine ausreichende bakterizide Wirkung von mindestens 5 lg wurde mit beiden Methoden in 16 Datensätzen von insgesamt 24 Datensätzen (je als Mittelwert der Dreifachbestimmung) gefunden, eine unzureichende bakterizide Wirkung von <5 lg wurde mit beiden Methoden in 7 Datensätzen gefunden. In einem Datensatz lag die mittlere lg-Reduktion mit der Mikromethode über 5 lg und mit der VAH-Methode unter 5 lg, wobei kein signifikanter Unterschied bestand (p=0,3096; 0,2% Glutaral, 1 min, *P. aeruginosa*). Eine ausreichende levurozide Wirkung von mindestens 4 lg wurde mit beiden Methoden in einem Datensatz gefunden, eine unzureichende levurozide Wirkung von weniger als 4 lg wurde mit beiden Methoden in 8 Datensätzen gefunden. Unterhalb der Wirksamkeitsgrenze konnten mit einer Ausnahme keine signifikanten Unterschiede der beiden Methoden festgestellt werden.

**Fazit:**

Die Mikromethode des quantitativen Suspensionsversuchs lieferte in 32 von 33 Versuchsansätzen identische Ergebnisse wie die VAH-Methode 9, wenn die bakterizide und levurozide Wirkung von Glutaral im Hinblick auf das Erreichen- oder Nichterreichen der Wirksamkeitsgrenze bewertet wird. Die Eignung der Methode sollte mit zusätzlichen Bakterienspezies, weiteren bioziden Wirkstoffen und in zusätzlichen Labors bestätigt werden.

## Introduction

For hand antiseptics as well as surface and instrument disinfectants used in healthcare settings in Germany, bactericidal and yeasticidal activity is a minimum requirement [1]. Efficacy is commonly demonstrated in quantitative suspension tests such as EN 13727 [2], EN 13624 [3] or the VAH method 9 [4], followed by tests simulating practice conditions. The quantitative methods yield comparable results [5], but require substantial amounts of laboratory material. In order to carry out a suspension test according to VAH method 9, for example with 3 concentrations and 3 contact times, a total of 79 agar plates, 162 mL of neutralizer and 32 mL diluent are necessary. As VAH attempts to encompass aspects of ecological sustainability in its efforts for quality assurance of disinfectants, the avoidance of waste, which includes laboratory waste during efficacy testing, has become a major concern. Thus, a microscale suspension test was developed to reduce the number of agar plates, neutralizer and diluents in the test procedure. In addition, a microscale suspension test could be used for a rapid assessment of disinfectant efficacy in field conditions. The aim of the present study was to determine whether the VAH method 9 and the newly developed microscale suspension test yield comparable reductions of the test organisms at various contact times to demonstrate the bactericidal and yeasticidal activity of glutaral at different concentrations.

## Methods

### Biocidal active substance and neutralizers

Glutaral (CAS number: 111-30-8) was used in this study and obtained from Sigma-Aldrich (Taufkirchen, Germany, Ref 340855, Batch number STBJ8154) at a concentration of 50% w/w in water. The concentration of the glutaral test solution was prepared to be 1.25 times higher than the concentration in the final suspension, because it is diluted by the addition of the organic load and test suspension. Glutaral was diluted in water of standardized hardness (WSH). The pH values were measured, revealing mean findings of 7.26 at a 0.05% concentration, 7.27 at 0.1%, 7.16 at 0.2%, and 7.09 at 0.3%. The following substances were used as neutralizers: polysorbate 80 (30 g/L), lecithin (9 g/L), L-histidine (1 g/L) and glycine (10 g/L). The suitability of the neutralizers was shown for each test organism according to VAH methods 9.1.6.1.2 and 9.1.6.1.3.

### Test organisms and test suspension

For bactericidal activity tests, *S. aureus* ATCC 6538 and *P. aeruginosa* ATCC 15442 were used. To obtain the test suspension, test organisms were cultured on tryptone soya agar (TSA). After 24 hours incubation at 37°C, the colonies were washed off with 10 mL dilution fluid (DF) tryptone-sodium chloride and mechanically suspended in DF for 3 min using 5 to 10 g of sterile glass beads with a diameter of 3 to 4 mm. The number of cfu in the test suspension was adjusted to 1.5–5.0x10^8^ per mL with DF using photometric control.

*C. albicans* ATCC 10231 was used to determine the yeasticidal activity. In order to obtain the test suspension, *C. albicans* was cultured on malt extract agar (MEA). After 48 h incubation at 30°C, the colonies were suspended with 10 mL of DF and mechanically suspended in DF for 3 min using 5 to 10 g of sterile glass beads with a diameter of 3 to 4 mm. The number of cfu in the test suspension was adjusted to 1.5–5.0x10^7^ per mL with DF using photometric control.

### VAH method 9

1.0 mL of the test suspension was mixed with 8.0 mL of the glutaral solution diluted in water of standard hardness and 1 mL of organic load (clean conditions – 0.3 g/L bovine albumin fraction V). After an exposure time of 1, 5 or 15 min, an aliquot of 0.5 mL of the mixture was transferred into 4.5 mL of TSB with neutralizers. Immediately thereafter, two serial 1:10 dilutions were prepared. After a neutralization time of at least 5 min ±10 s, aliquots of 1 mL (product-neutralizer mixture) and 0.1 mL (product-neutralizer mixture and both dilutions) were plated on TSA (bacteria) or MEA (*C. albicans*). Plates were incubated for 48 h at 36°C (bacteria) or for 72 h at 30°C (*C. albicans*), followed by cfu counting. The reduction of micro-organisms was calculated as the difference between the lg cfu of the water control (no disinfectant) and the lg cfu of the glutaral-treated sample after the exposure time.

### Microscale suspension test

**Figure 1:**
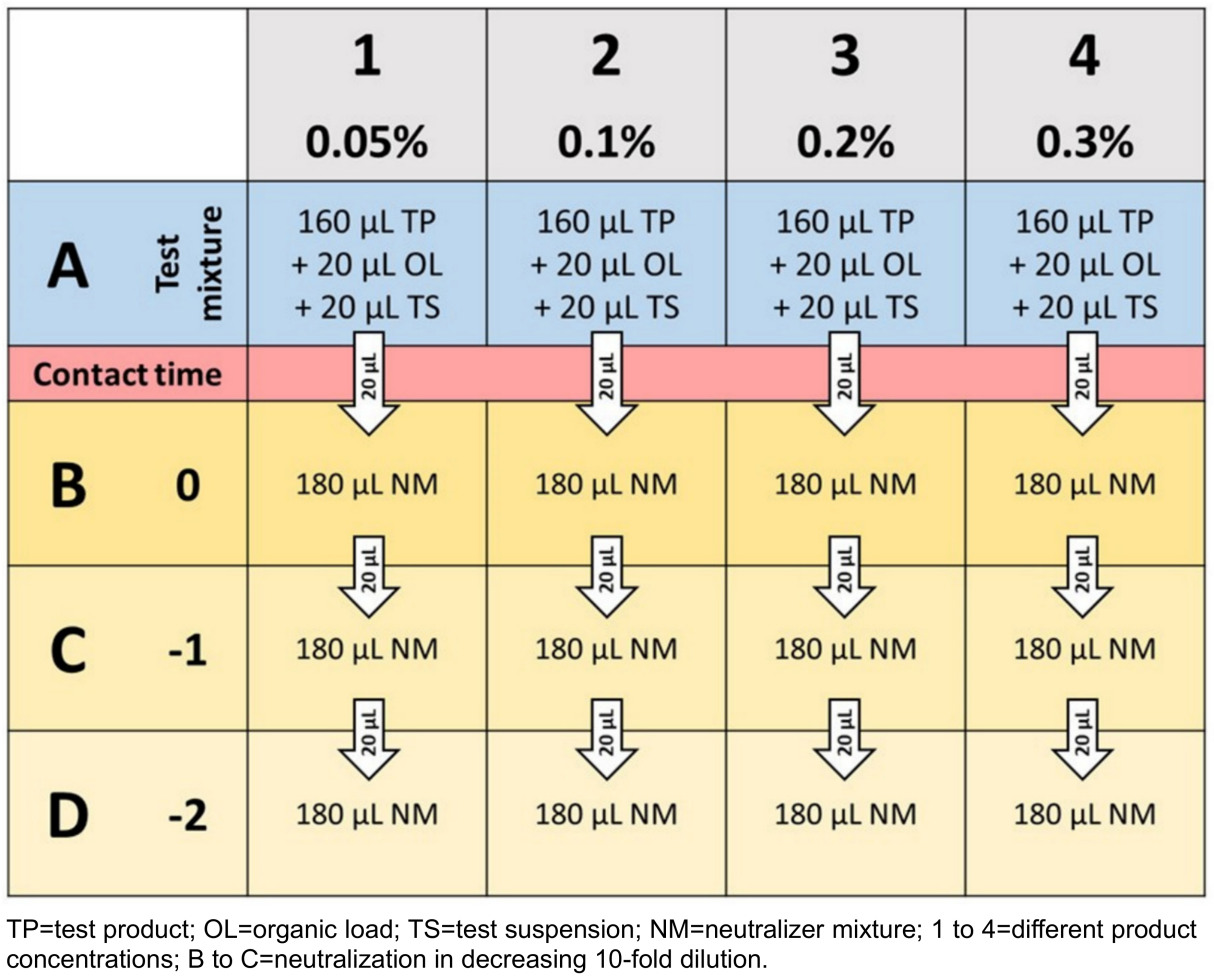
Illustration of the main steps of the microscale suspension test

The tests were performed in separate 96-well plates for each test organism (Roth, Karlsruhe Germany, Rotilabo – flat bottom – 345 µL). The different test compounds (different concentrations of the product test solutions and water controls) were allocated to the vertical rows 1 to 12 of the 96-well plate, the serial dilutions for the different contact times were allocated to the horizontal rows from A to H. An example for a test setup with a product with 4 different concentrations (vertical rows 1 to 4) and one contact time with 3 dilution steps (horizontal rows A to D) is given in Figure 1 (Fig. 1).

The experiments were carried out with product test solutions containing 0.3 g/L bovine albumin fraction V, which was added immediately prior to the start of the test. For this purpose, 160 µL of the product test solution was mixed thoroughly with 20 µL of a 3 g/L bovine albumin fraction V solution and immediately with 20 µL of the test suspension (Figure 1 (Fig. 1)). The first well (A1) was filled with 160 µL of the respective test product dilution and 20 µL of the loading substance. The same volumes were added to wells A2, A3 and A4, with increasing concentration of the test product dilution (e.g. 0.1%, 0.2%, 0.3%). For the water control (A5), water of standardized hardness (WSH) was added instead of the test product. The same test procedure was performed as reproductions in the same microtiter plate in columns 6–10. 180 µL of neutralizer was added to each of the wells B1 to H10.

**Figure 2:**
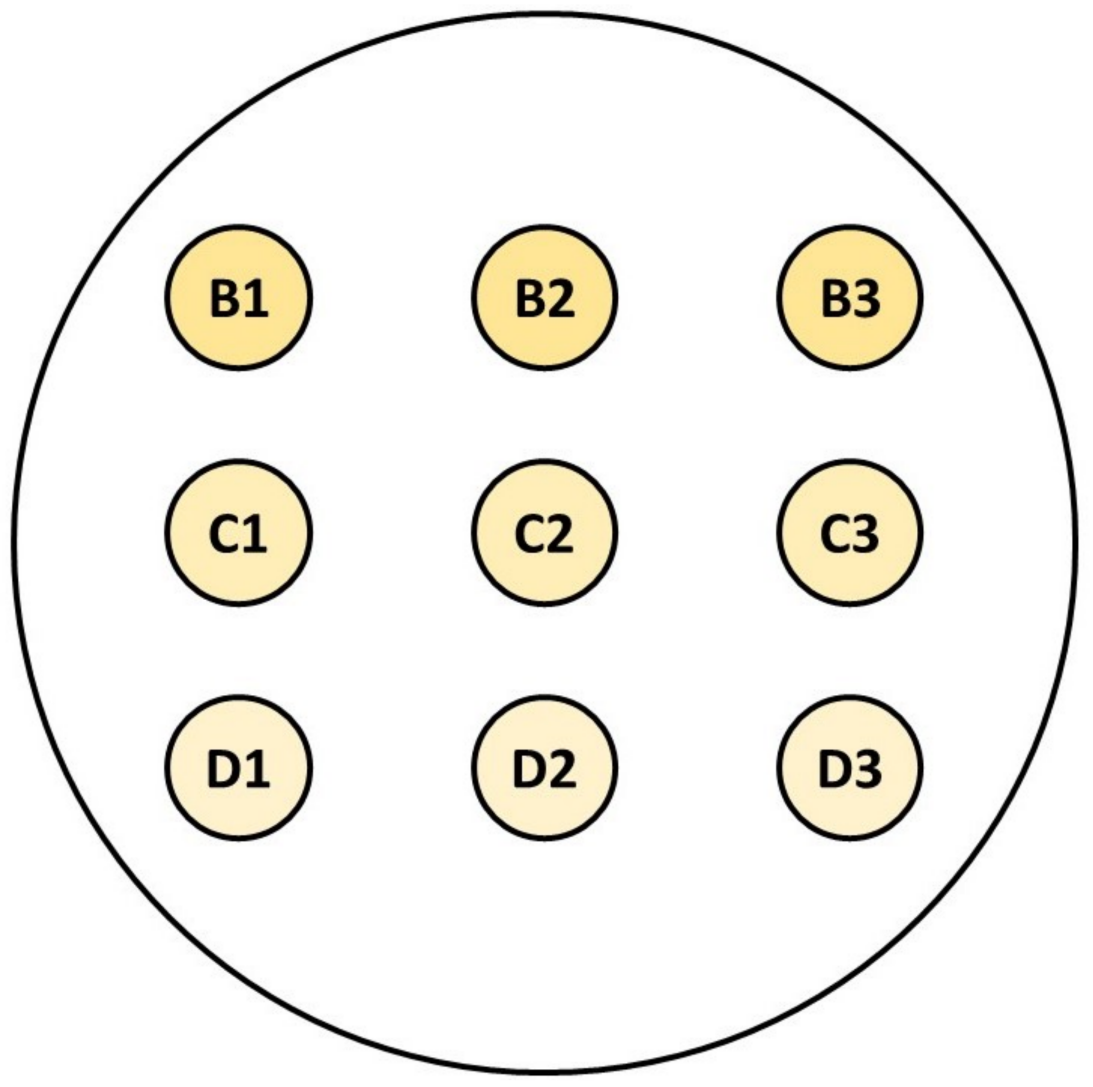
Example for placing the 10 µL aliquots of neutralizing mixture on solid agar plates using the microscale suspension test method

At time t0, 20 µL of test suspension was pipetted into the wells of row A using a multichannel pipette, and the test wells were mixed by repeated aspiration with the multichannel pipette with new pipette tips (Figure 1 (Fig. 1)). At the end of the exposure time (t_1min_, t_5min_ and t_15min_), 20 µL of the test preparation were pipetted into each well and neutralized. The neutralization mixture was mixed by repeated aspiration of the solution with the multichannel pipette with new pipette tips. For the shortest exposure time, two additional dilution steps (rows C and D) were added to the direct preparation (row B), and for the two longer exposure times, only one additional dilution step (row F or H) was added to the direct preparation (row E or G). After a neutralization time of at least 5 min ±10 s, 100 µL of the first neutralization mixture and 10 µL of each neutralization mixture were pipetted onto CSA (*C. albicans*: MEA) culture media for cfu determination, then allowed to dry as a flat drop without spreading the mixture. Depending on the preparation, up to 9 aliquots were placed on one culture dish with one and the same culture medium (Figure 2 (Fig. 2)).

Priority was given to the evaluation of agar plates in which the number of cfu was between 1 and 100 (*S. aureus* and *P. aeruginosa*) or between 1 and 50 (*C. albicans*). The cfu count was performed macroscopically without any optical magnification. The reduction R was calculated according to the following formula:

Lg R=lg (cfu Co1)–lg (cfu D)

where cfu Co1 was the number of cfu per ml without exposure to the product (water control) and cfu D was number of cfu per ml after exposure to the product.

For the water control (Co1), WSH was used instead of glutaral. After the contact time, it was transferred to the neutralizer (series B) and diluted to 10^–4^ (*C. albicans*: 10^–3^) in series C to F. From the neutralization mixtures at dilutions 10^–2^ to 10^–4^ (*C. albicans*: 10^–2^ to 10^–3^), 10 µL were pipetted onto a culture medium for CFU determination.

For the neutralization control (Co2), 20 µL of the highest concentration of the test product used in the test was mixed with 180 µL of neutralizing agent at 20°C and, after a neutralization time of 10 s±1 s, 2 µL of a 10^–3^ dilution of the CFU determination series (*C. albicans*: 10^–2^ dilution) of the test suspension was added. After the longest exposure time, 10 µL of this was pipetted onto CSA (*C. albicans*: MEA), both from the direct preparation and from a 10^–1^ dilution in neutralizing agent.

The neutralizing agent non-toxicity control (Co3) was performed in parallel with control Co2, but contained WSH instead of the product test solution. After adding the diluted test suspension and a 5 min ±10 s incubation time at 20°C, 10 µL of this was pipetted onto CSA (*C. albicans*: MEA), both from the direct preparation and from a 10^–1^ dilution in neutralizing agent. 

Columns 11 and 12 of the microtiter plate were used to determine the concentration of the initial suspension N. For this purpose, 180 µL of dilution fluid (DF) tryptone-sodium chloride was added to each of the wells A11-G11 and A12-G12. The test suspension was added to each of wells A11 and A12 and mixed thoroughly; then 20 µL was pipetted into B11 and B12. The dilution steps were continued until 10^–7^ (*C. albicans*: 10^–6^). From dilutions 10^–4^ to 10^–7^ (*C. albicans*: 10^–3^ to 10^–6^), i.e., rows D to G (*C. albicans*: rows D to F), 10 µL of each well was pipetted onto CSA (*C. albicans*: MEA).

Each experiment was carried out in triplicate. Mean values obtained with both methods were compared using the unpaired t-test. A p-value <0.05 was considered to be significant.

## Results

### Bactericidal and yeasticidal activity

**Figure 3:**
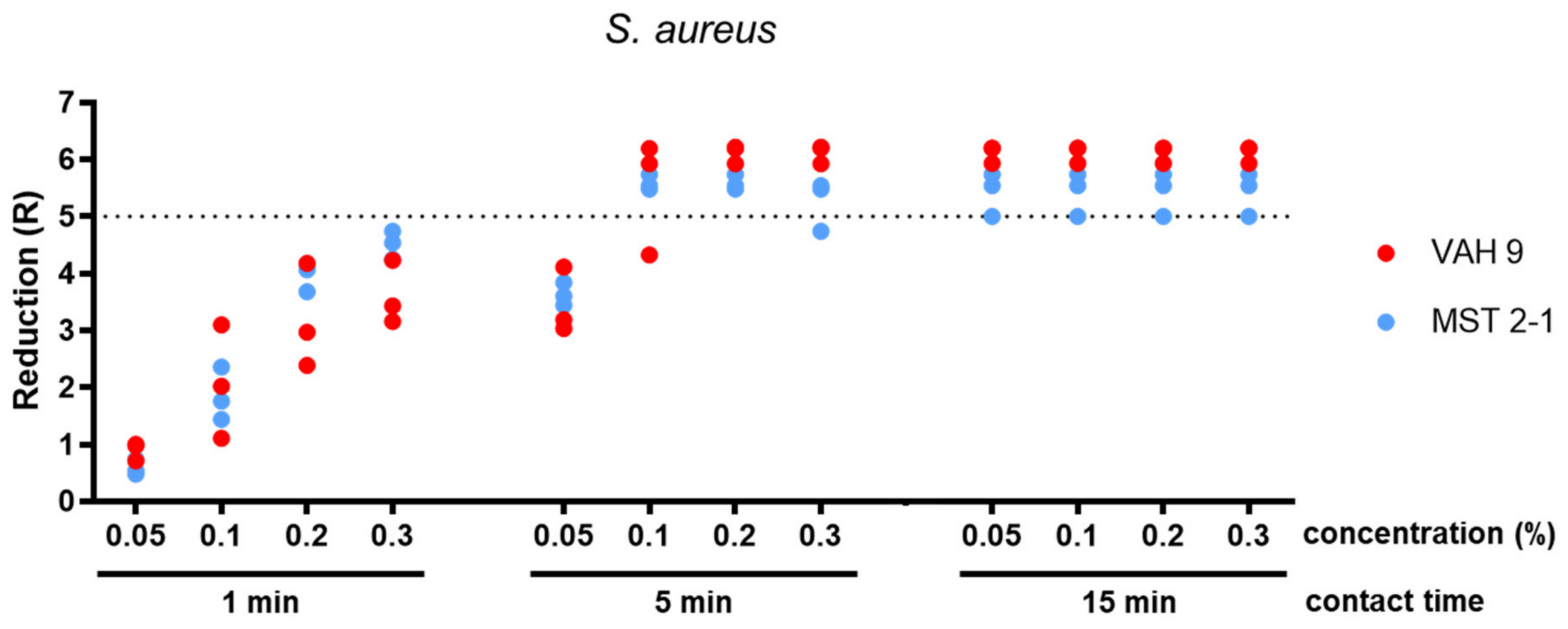
Reduction for glutaral concentrations 0.05%, 0.1%, 0.2% and 0.3% at the contact times 1 min, 5 min, and 15 min against *S. aureus*. Means from three independent biological replicates according to the microscale method (MST 2-1) and VAH method (VAH 9).

**Figure 4:**
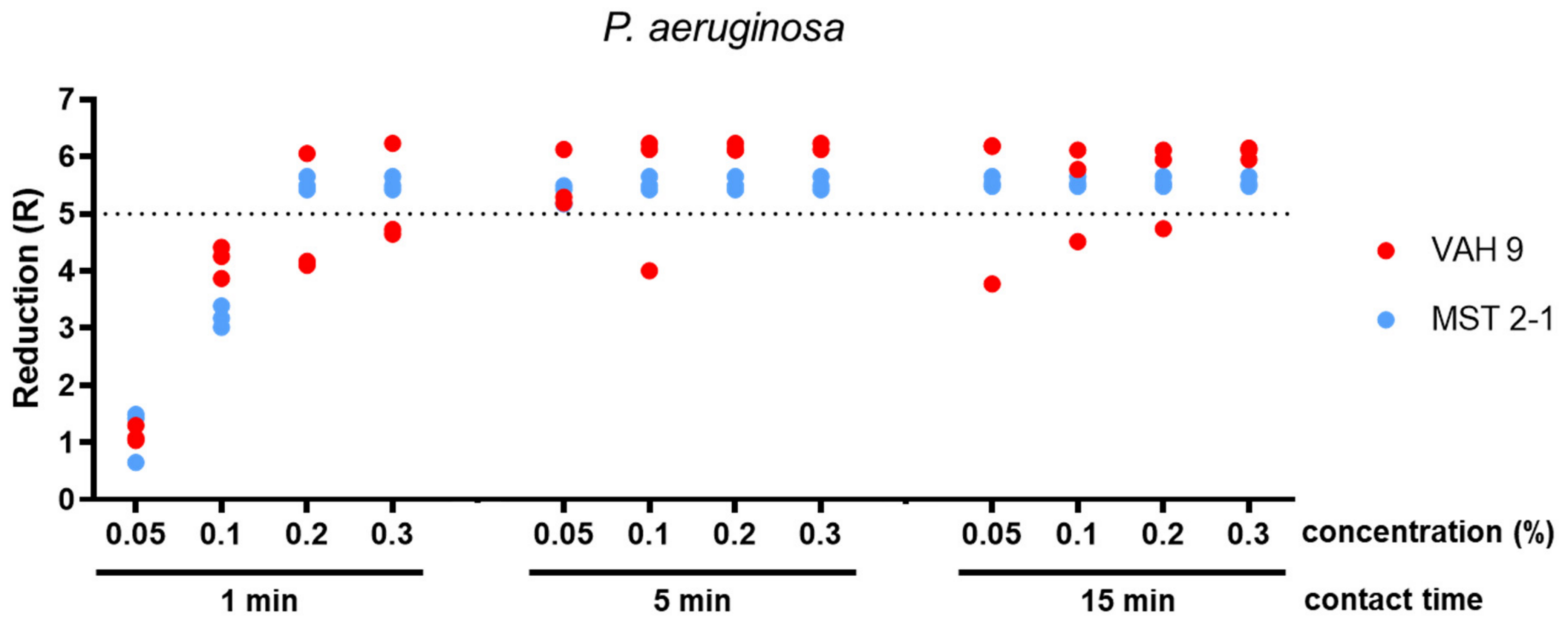
Reduction for glutaral concentrations 0.05%, 0.1%, 0.2% and 0.3% at the contact times 1 min, 5 min, and 15 min against *P. aeruginosa*. Means from three independent biological replicates according to the microscale method (MST 2-1) and VAH method (VAH 9).

**Figure 5:**
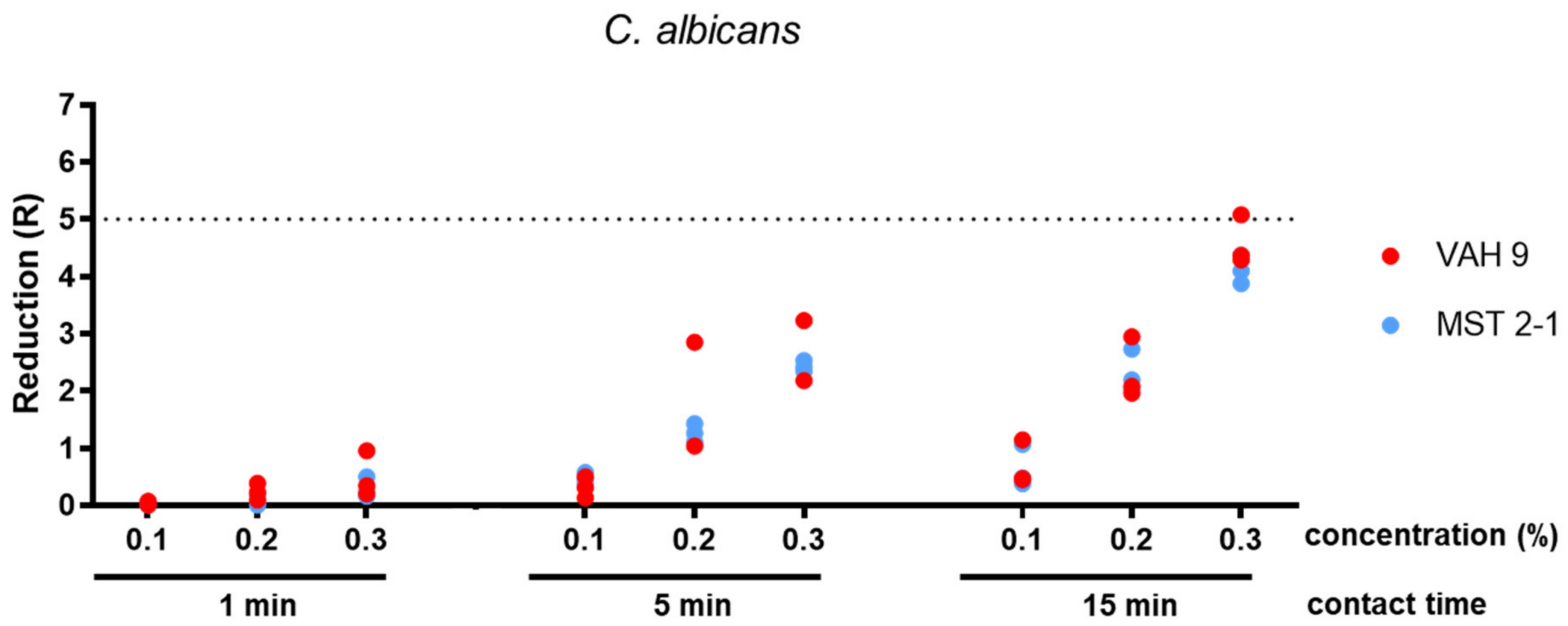
Reduction for glutaral concentrations 0.1%, 0.2% and 0.3% at the contact times 1 min, 5 min, and 15 min against *C. albicans*. Means from three independent biological replicates according microscale method (MST 2-1) and VAH method (VAH 9).

The concentration of 0.05% glutaral was tested only against *S. aureus* and *P. aeruginosa*. After 1 min, the mean reduction was below 5 lg for both species. After 5 min, it was at least 5 lg for *P. aeruginosa* and <5 lg for *S. aureus*. The 15 min exposure time yielded sufficient bactericidal activity against both *S. aureus* and *P. aeruginosa* (both methods; Table 1 (Tab. 1), Figure 3 (Fig. 3), Figure 4 (Fig. 4), Figure 5 (Fig. 5)). 

A concentration of 0.1% glutaral was not sufficiently effective within 1 min against all three test organisms. After a 5 min exposure, the mean reduction was at least 5 lg for *P. aeruginosa* and for S*. aureus* (both methods). However, it was <4 lg with *C. albicans* (both methods). When exposed for 15 min, the mean lg reductions were at least 5 for both bacterial species and <4 lg for *C. albicans* (both methods; Table 1 (Tab. 1), Figure 3 (Fig. 3), Figure 4 (Fig. 4), Figure 5 (Fig. 5)).

Glutaral at 0.2% was not able to reduce *S. aureus* and *P. aeruginosa* by at least 5 lg within 1 min (both methods) or *C. albicans* by at least 4 lg (both methods). The 5 min exposure time yielded sufficient bactericidal activity against *S. aureus* (both methods) and for *P. aeruginosa* (only with the microscale method; p=0,310). With an exposure time of 15 min, however, *S. aureus* and P*. aeruginosa* were reduced by at least 5 lg (both methods), whereas *C. albicans* was reduced by less than 4 lg (both methods) (Table 1 (Tab. 1), Figure 3 (Fig. 3), Figure 4 (Fig. 4), Figure 5 (Fig. 5)).

A solution of 0.3% glutaral reduced *P. aeruginosa* sufficiently within 1 min, but not *S. aureus* or *C. albicans* (both methods). When exposed for 5 min, however, both bacterial species were reduced by at least 5 lg (both methods), whereas *C. albicans* was reduced by less than 4 lg (both methods). After 15 min, the yeasticidal activity was also sufficient, with at least 4 lg (both methods; Table 1 (Tab. 1), Figure 3 (Fig. 3), Figure 4 (Fig. 4), Figure 5 (Fig. 5)).

The differences between the means obtained with both methods were significant in 11 of 33 data sets (p<0.05; Table 1 (Tab. 1)). In all eleven of them, however, the overall results remained the same when, for example, the means of both methods were above (n=10) or below (n=1) the efficacy threshold. In none of the 33 data sets was the difference between the means significant, resulting in a mean below the requirement level with the one method and a mean above the requirement level with the other method (Table 1 (Tab. 1)). The significant differences in reduction may be attributed to the different detection limits of the two methods, due to the different sample volume of 0.1 ml as the maximum for the microscale method (detection limit <6 lg) and 0.5 ml as the maximum for the VAH method (detection limit >6 lg). With one exception, no significant difference was found between the two methods below the efficacy threshold (Table 1 (Tab. 1)).

### Consumption of agar plates and media

For all experiments done according to VAH method 9, a total of 711 agar plates, 486 mL of neutralizer and 95 mL of diluent were used. The material consumption was lower when the microscale method was used, with a total of 126 agar plates (82.3% less), 58 mL of neutralizer (96% less) and 11 mL of diluent (96% less).

## Discussion

There are a number of research publications on microscale suspension tests, however, none of them have evaluated their reliability in determining the microbiocidal activity of disinfectants. Traoré et al. [6] determined the bactericidal activity of povidone iodine in a microscale suspension test, but without a neutralization step and with a very small aliquot of 1.5 µl transferred to the agar plate, potentially yielding a greater variation of results. Three other research teams applied a semiquantitative microscale suspension test without spreading inocula on solid surface agar, so that uncertainty remains as to whether the test organism has caused the visible growth in the test tubes [7], [8], [9]. We were able to show that the results of our microscale suspension test were comparable overall in terms of the bactericidal and yeasticidal activity of glutaral to those of VAH method 9. Although data for other biocidal agents and other test organisms are still lacking, our findings using glutaral at different concentrations and with *S. aureus* and *P. aeruginosa* as test organisms indicate that this microscale suspension test will provide results similar to those obtained with a standard suspension test.

The VAH method (former DGHM method) has been previously compared with other European standard scale suspension tests (prEN 12054), e.g., with three alcohol-based hand rubs [10]. They yielded comparable results for the standard test bacteria, suggesting that there were no major technical differences between both methods. 

One major advantage of the microscale suspension test is lower consumption of laboratory materials, such as agar plates (82.3% less), neutralizer (96% less) and diluent (96% less). It can therefore be regarded as a step towards improved sustainability in disinfectant testing. In addition, the microscale method contributes to saving time, energy, and incubator capacity. Laboratories are called upon to use resources efficiently and responsibly with the aim to improve sustainability in health care [11]. The microscale suspension test can be considered a step in this direction. European standards which could be performed by the microscale suspension test are listed in Table 2 (Tab. 2). It must be mentioned, however, that at present, no statements can be made on the suitability of the method with regard to fungicidal activity or disinfectant products, which often consist of various active substances. 

Another major advantage of the microscale suspension test is the possibility of showing the threshold between effective and ineffective concentrations and times with countable cfu. The microscale suspension test is therefore suitable for conducting screening tests for testing a large number of isolates with regard to possible tolerances or resistances against disinfectants.

A limitation of our work is that the results were obtained in only one laboratory. It was already described in 1977 that suspension tests may yield variable results in different laboratories [12]. In 1979, it was shown that conducting the same suspension test in different laboratories can yield significantly different results, although the authors indicated that the variance was rather low for this type of experiment [13]. Based on results on the repeatability of suspension tests [14], it became a major aim to define as many technical details as possible in order to avoid relevant differences in the execution of the suspension tests between laboratories (e.g. in EN 13727 or VAH method 9). Bearing the results on variability in mind, it seems to be a relevant next step to evaluate the microscale suspension test in other laboratories, e.g., as a VAH-coordinated ring trial with multiple laboratories.

In 2023, the VAH organized an interlaboratory test for a quantitative suspension test according to EN 13727. Thirty-one laboratories participated in this ring trial. *S. aureus* was chosen as the test organism and glutaral was used as the test product in the concentrations of 0.01%, 0.05% and 0.1% at 5 min under low organic load. On average, reductions ranged from 0.345±0.123 lg (0.01%), to 4.155±0.337 lg (0.05%) and 5.293±0.094 lg (0.1%) (preliminary data, as yet unpublished). The data obtained in the present study with the microscale method using *S. aureus* at 5 min contact time were 3.62±0.20 lg for 0.05% and 5.58±0.14 lg for 0.1%, and are thus comparable with the pertinent concentration/time relations of the VAH ring trial. 

Another limitation of our study is that the only biocidally active substance evaluated was glutaral. In further studies, it must be shown that different biocidally active substances and/or biocidal products based on biocidally active substances, such as peroxides, alcohols or quaternary ammonium compounds, will also lead to reliable results.

A further limitation is the limited number of test organisms. EN 13727 requires using three bacterial species to determine the bactericidal activity of disinfectants [2]. In the present study, only two were used (*S. aureus* and *P. aeruginosa*). It is therefore possible that the results may be different with the remaining test strain *E. hirae*. Future research will have to show whether the promising results of our study can be consolidated with other test strains or with clinical isolates. The glutaral solutions we used had an acidic pH below 7.3, conferring stability but lower bactericidal activity. Thus, our results may not be directly applicable to the more potent, alkalized solutions available commercially.

## Conclusions

The microscale suspension test yielded results similar to those of VAH method 9 when the bactericidal and yeasticidal activity of glutaral were evaluated. Its suitability should be confirmed with *E. hirae*, additional biocidally active substances and in additional laboratories.

## Notes

### Conflicts of interest

Günter Kampf received honoraria from Schülke & Mayr, Germany, outside the submitted work. The other authors declare no conflict of interest. The views expressed here are those of the authors and do not necessarily reflect those of the universities or institutions they are affiliated with.

## Figures and Tables

**Table 1:**
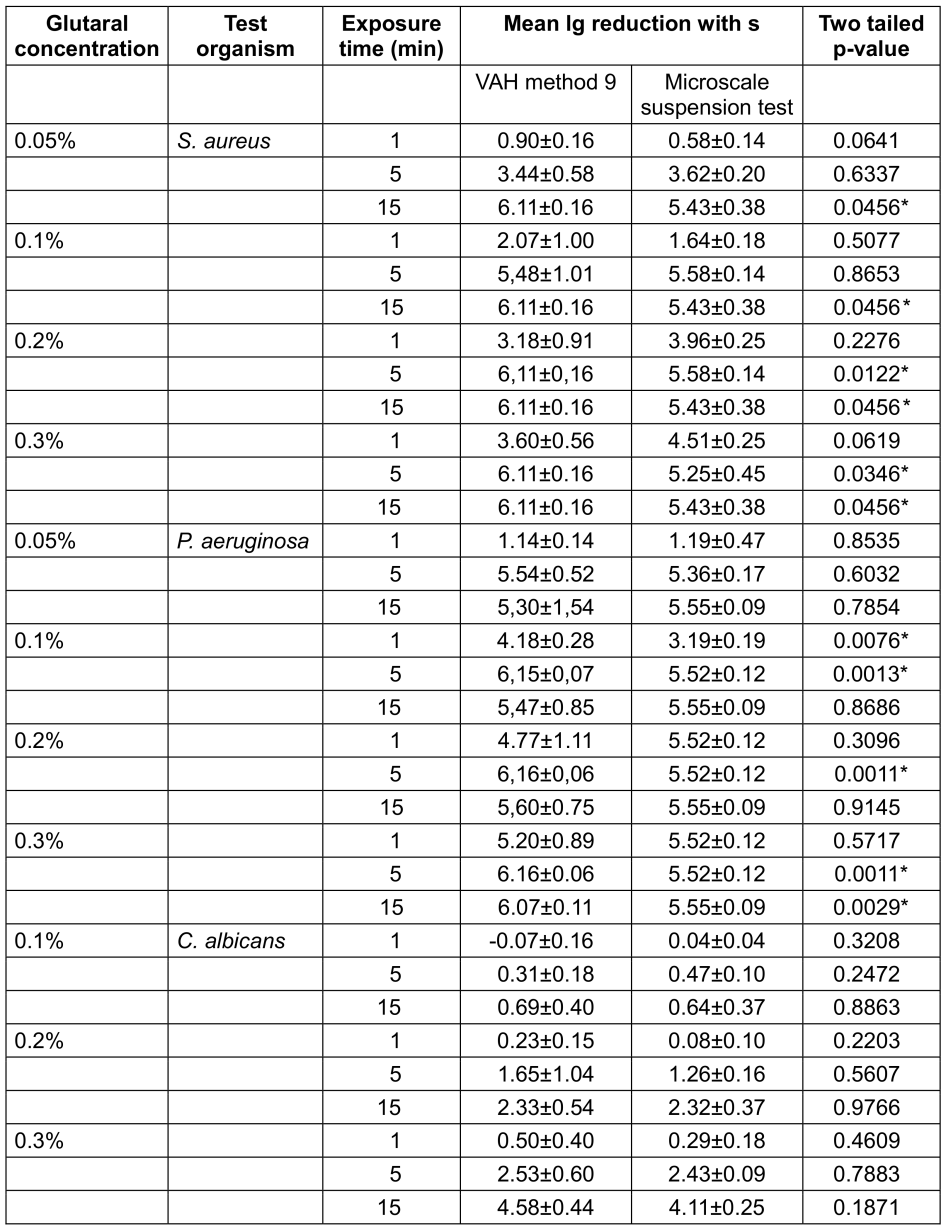
Mean lg reduction with standard deviation (s) obtained with glutaral at different concentrations and exposure times in two types of suspension test against *S. aureus*, *P. aeruginosa* and *C. albicans*; results are based on three experiments; p-values were obtained using the unpaired t-test. *=significant atp <0.05.

**Table 2:**
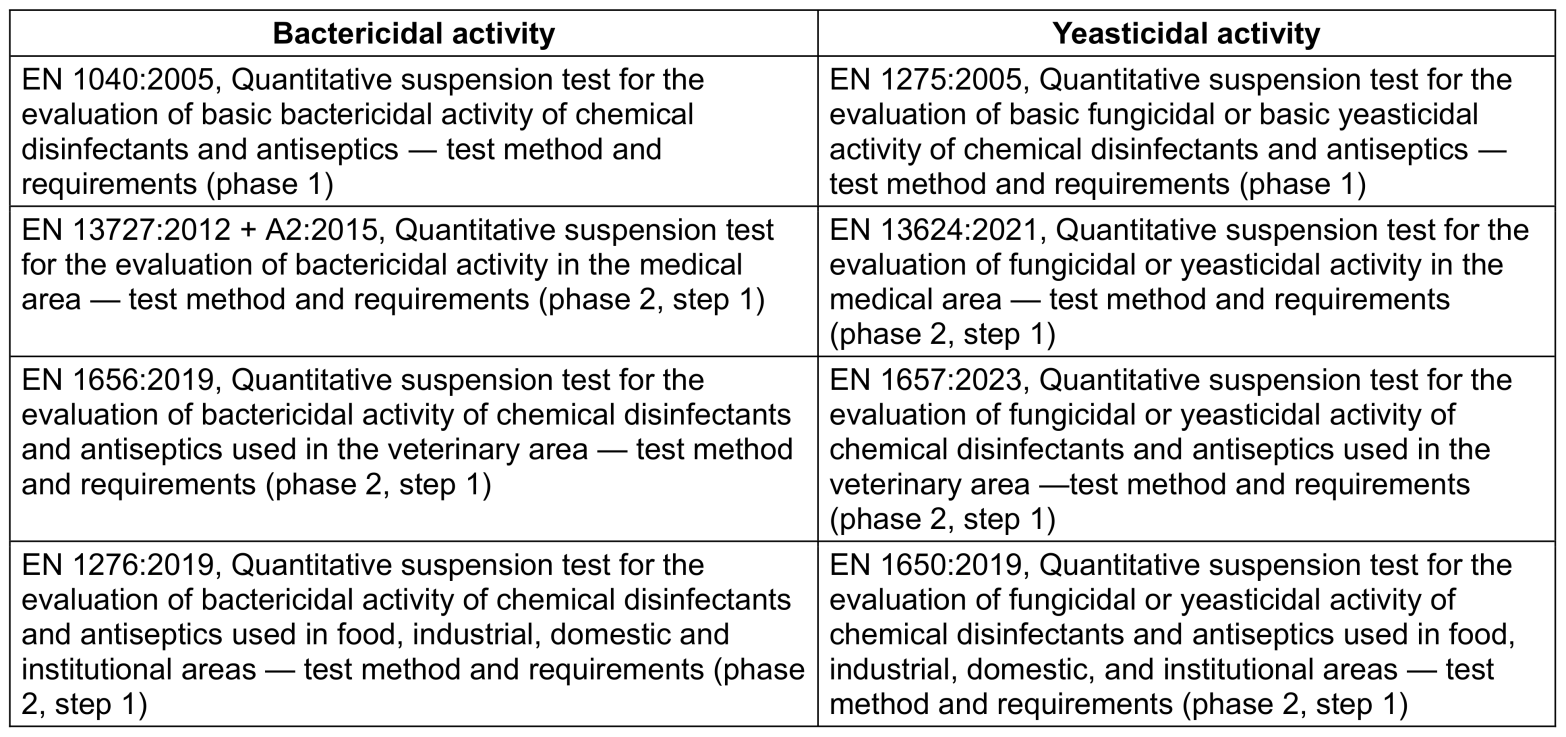
Overview of European standards for testing chemical disinfectants and antiseptics suitable for a microscale method
